# Short-range order of compressed amorphous GeSe_2_

**DOI:** 10.1038/srep10188

**Published:** 2015-05-14

**Authors:** L. Properzi, A. Di Cicco, L. Nataf, F. Baudelet, T. Irifune

**Affiliations:** 1 Physics Division, CNISM, School of Science and Technology, Università di Camerino, I-62032 Camerino (MC), Italy; 2 Synchrotron Soleil, L’Orme des Merisiers, St. Aubin 91192 Gif s/Yvette, France; 3 Geodynamics Research Center, Ehime University, Matsuyama 790-8577, Japan; 4 Earth-Life Science Institute, Tokyo Institute of Technology, Tokyo 152-8550, Japan

## Abstract

The structure of amorphous GeSe_2_ (a-GeSe_2_) has been studied by means of a combination of two-edges X-ray absorption spectroscopy (XAS) and angle-dispersive X-ray diffraction under pressures up to about 30 GPa. Multiple-edge XAS data-analysis of a-GeSe_2_ at ambient conditions allowed us to reconstruct and compare the first-neighbor distribution function with previous results obtained by neutron diffraction with isotopic substitution. GeSe_2_ is found to remain amorphous up to the highest pressures attained, and a reversible 1.5 eV red-shift of the Ge K-edge energy indicating metallization, occurs between 10 GPa and 15 GPa. Two compression stages are identified by XAS structure refinement. First, a decrease of the first-neighbor distances up to about 10 GPa, in the same pressure region of a previously observed breakdown of the intermediate-range order. Second, an increase of the Ge-Se distances, bond disorder, and of the coordination number. This stage is related to a reversible non-isostructural transition involving a gradual conversion from tetra- to octa-hedral geometry which is not yet fully completed at 30 GPa.

Tetrahedral systems such as Si/Ge, ice, silica, germania and chalcogenides have been playing a key role in the study of disordered systems due to their open structure which favors the presence of several polymorphs with different packing conditions. Besides this fundamental interest, chalcogenides show one of the highest glass-forming capabilities of the periodic table, which paves the way to many applications such as infra-red lenses and phase-change memories.

The main difference between GeSe_2_ and the archetypal tetrahedral glass forming oxides (SiO_2_, GeO_2_) is the presence of edge-sharing polyhedra^1^. This is due to a reduction of the mean Ge-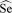-Ge bond angle below the tetrahedral one (109.5°) and therefore to a distortion of the Ge-Se_4_ structural units^2^. Presence of chemical disorder was found in the glass structure both by neutron diffraction (isotopic substitution)^1^,^3^ and by computer simulations^4^.

Recently, the properties of GeSe_2_ under high pressure have been studied in its liquid phase up to 5.1 GPa, evidencing the disruption of intermediate-range order by a reduction of the first-sharp diffraction peak (FSDP)^5^ intensity. The interest for this substance is enhanced by the presence of a null-derivative point of the melting line around 1 GPa, which epitomizes the competition between two different packing conditions and which can be directly connected to the pressure-induced amorphization of its crystalline counterpart at room temperature^6^. Furthermore, acoustic measurements have been carried out up to 9.6 GPa, showing a gradual structural transition in the glass at about 4 GPa^7^. This has been related to the presence of two different compaction mechanisms: the conversion of edge-sharing tetrahedra to corner-sharing geometry at low pressures and an increase of the average coordination number at higher pressures. The mean Ge coordination number has been measured to increase to about 4.5 at 9.3 GPa by neutron and x-ray diffraction^8^ (XRD). Moreover, molecular-dynamics simulations have suggested a gradual transition to sixfold Ge coordination, associated with the closure of the optical band-gap^4^. Nevertheless, the structure of amorphous GeSe_2_ for pressures above 10 GPa has not been investigated experimentally and no evidence for its pressure-induced metallization has been provided. Moreover, the debate over the presence of a continuous rather than a broken bond network is still open^9^.

X-ray absorption spectroscopy (XAS) represents a suitable technique for investigating the evolution of the local structure under pressure, ideally complementary to X-ray and neutron diffraction which are more sensitive to medium and long-range ordering. An additional advantage is that XAS is element-specific and allows for easier collections of data sets related to different chemical species in multi-atomic substances. In this work, the short-range atomic structure of amorphous germanium diselenide (a-GeSe_2_) is studied at both Ge and Se atomic sites by performing two-edges high-pressure XAS experiments up to 30 GPa. The main aim of this study is providing new and detailed short-range structural information of the glass at high-pressure shedding light on the transformations occurring during densification. The use of multiple-edge XAS refinement^10^, corroborated by simultaneous high-pressure XRD measurements, is intended to provide reliable structural data and identify possible crystallization phenomena.

## Results

**Figure 1:**
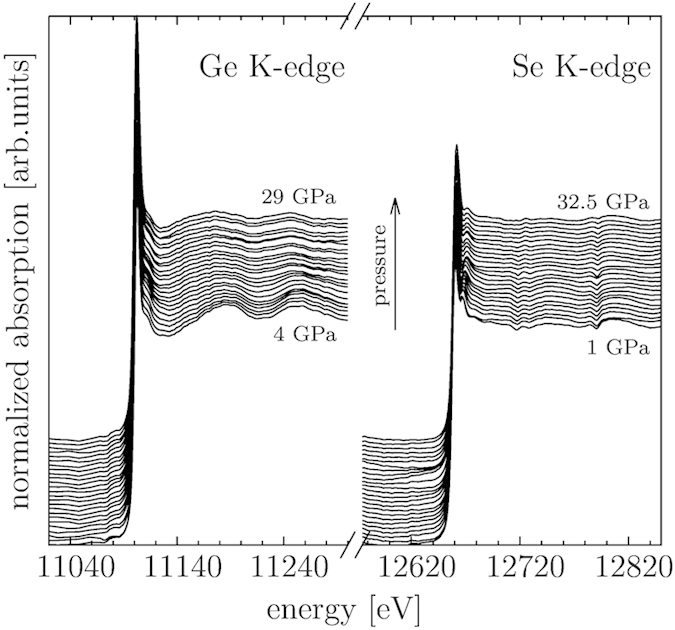
XAS spectra of a-GeSe_2_ for increasing pressure collected using nano-polycrystalline diamonds (NPD)^14^,^15^. Ge and Se K-edge XAS spectra are reported on the left and right-side of the figure respectively.

High-quality Ge and Se K-edge XAS spectra of crystalline (c-GeSe_2_) and amorphous (a-GeSe_2_) germanium diselenide were measured in scanning energy mode at the XAFS beamline^11^ at ELETTRA (Trieste, Italy), with the intent of providing a benchmark for the high-pressure XAS measurements in dispersive mode successively performed at the ODE beamline^12^,^13^ shown in Fig. 1.

Extended x-ray absorption fine structure (EXAFS) spectra of crystalline (c-GeSe_2_) and amorphous (a-GeSe_2_) germanium diselenide were analyzed by *ab initio* multiple-scattering calculations within a well established approach of EXAFS data analysis^16^,^17^,^18^. Within this approach, the structural refinement of EXAFS spectra provides direct information on the pair (and higher order) distribution functions. Multiple-edge structural refinement^10^ was performed for both Ge and Se K-edge EXAFS spectra in both c-GeSe_2_ and a-GeSe_2_.

**Figure 2:**
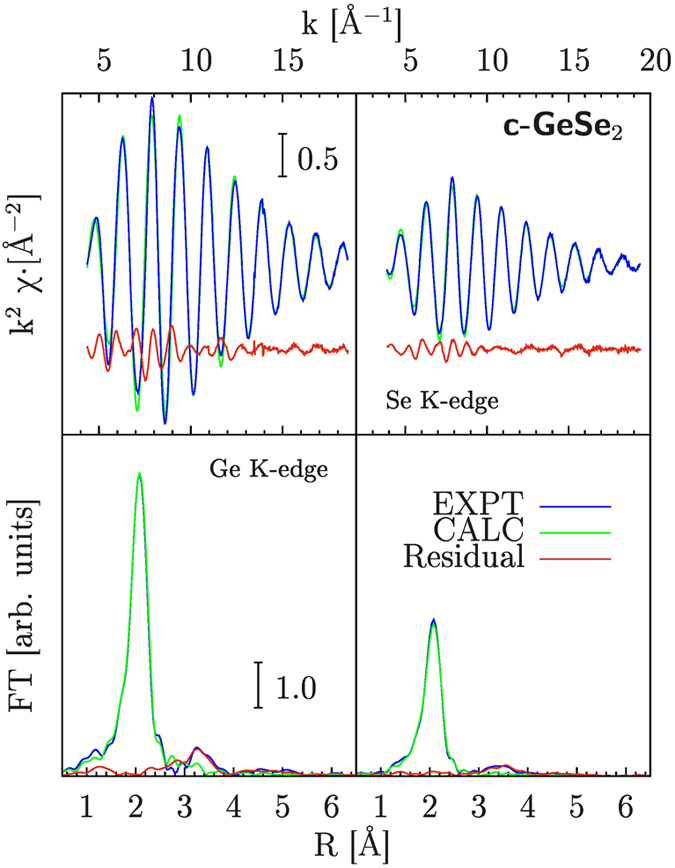
Ge (Left) and Se (Right) K-edge XAS spectra of crystalline GeSe_2_ (c-GeSe_2_). Experimental (EXPT) and best-fit calculations (CALC), accounting for the dominant signal associated with the first-neighbor Ge-Se distribution, are compared. The Fourier Transform (FT) of both signals showing a prominent peak at about 2 Å related to the Ge-Se first-neighbors, is compared in the lower panels. The differences between experimental and calculated spectra (Residual curves) are weak oscillating signals associated with more distant neighbor distributions (above 3 Å in the FT spectra).

As shown in Fig. 2, the first neighbor distribution related to the Ge-Se covalent bonding is associated with the dominant Ge-Se (Se-Ge) two-body signal reproducing most of the EXAFS spectral features. In Fig. 2, upper panels, the EXAFS 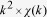 experimental and calculated best-fit signals for Ge (left) and Se (right) are compared. The weak high-frequency residual curves (lower curves) shows that in this crystalline material second and further shell atoms give a negligible contribution to the EXAFS spectra. The Fourier Transforms (FTs) of the EXAFS spectra (lower panels of Fig. 2) show a prominent peak at the typical Ge-Se first-neighbor distance (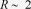  Å effective FT distance). The residual signals are mainly associated with a very weak contribution of the second and further neighbors (second neighbor Ge-Ge and Se-Se above 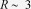 Å effective FT distance). The Ge and Se K-edge data are presented on the same vertical scale and the difference in the amplitude of the signal is mainly associated with the different coordination Ge-Se and Se-Ge numbers within this structure and with the backscattering amplitudes of Ge and Se.

The resulting best-fit first-neighbor structural parameters for c-GeSe_2_ were 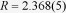 Å and 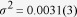 Å^2^ for the average distance and bond variance respectively (statistical errors quoted in brackets). The first-neighbor distribution was assumed to be Gaussian, with fixed coordination numbers (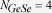, 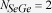) as resulting from the known crystal structure (see Ref. 6 and refs. therein). Introduction of skewness (non-Gaussian distributions, see for example Ref. 19) was not found to be statistically significant for improving the fitting.

**Figure 3:**
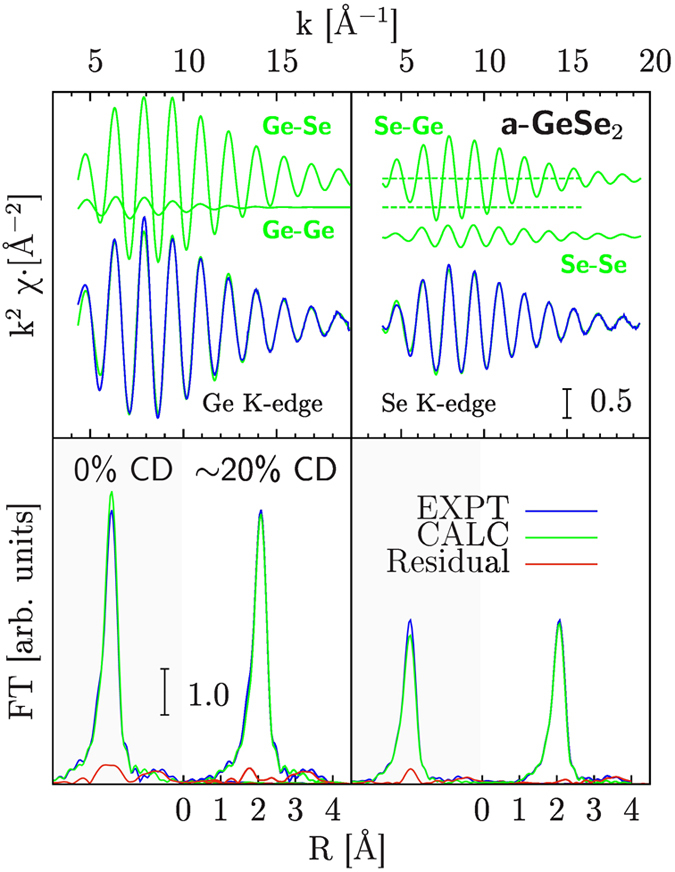
Ge (Left) and Se (Right) K-edge XAS spectra of amorphous GeSe_2_ (a-GeSe_2_). Experimental (EXPT) and best-fit calculations (CALC) are compared in both panels. The dominant Ge-Se first-neighbor signal is accompanied by weaker short-range Ge-Ge and Se-Se signals, due to the existence of chemical disorder. Those first-neighbor signals explain nicely the experimental data, being the medium-range ordering signal above 3 Å almost negligible as shown by Fourier Transform (FT) spectra of experimental, best-fit calculated, and residual signals in the lower panels. The bottom frames show also the fit done without chemical disorder (CD) in order to make the reader appreciate the improvement of the residue.

Our XAS structural refinement of the short-range structure of a-GeSe_2_ is reported in Fig. 3, taking into account the previous results obtained by neutron-diffraction^1^. The partial radial distribution functions obtained by neutron diffraction indicated occurrence of chemical disorder in a-GeSe_2_, leading to the formation of first-neighbor Ge-Ge and Se-Se bonds^1^,^3^. Accounting of chemical disorder in the XAS calculations has been performed allowing for the presence of first-neighbor Ge-Ge and Se-Se bonds with the constraint of keeping fixed the total coordination number (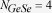, 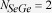) and stoichiometry. This was realized by introducing a chemical order parameter as a fitting parameter (*x*, with *x* = 0 for perfect chemical ordering). The improvement of the fit obtained by introducing chemical disorder was found to be statistically meaningful (see Fig. 3), while the introduction of further degrees of freedom (like floating total coordination numbers or allowing for slight variations of stoichiometry) was considered to be unjustified at ambient conditions. Individual Ge-Se (Se-Ge), Ge-Ge, and Se-Se XAS best-fit calculated signals are reported in Fig. 3 (upper curves), and the agreement of the total best-fit calculations with the experimental data for a-GeSe_2_ is found to be very good, for what concerns both the EXAFS signals (upper panels) and the Fourier Transforms (lower panels). A very weak residual signal (see lower panels) is associated with medium-range ordering within the glass. The resulting best-fit first-neighbor structural parameters for a-GeSe_2_ (estimated statistical errors quoted in brackets) were: 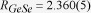 Å, 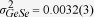 Å^2^, 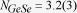 (Ge-Se first-neighbors); 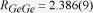 Å, 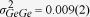 Å^2^, 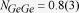 (Ge-Ge first-neighbors); 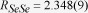 Å, 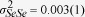 Å^2^, 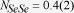, (Se-Se first-neighbors). The possibility of detection of chemical disorder is here mainly due to the strong constraints imposed by the double-edge refinement and by the extended 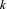-space fitting on raw data^10^. Average Ge-Se first-neighbor distance and bond variance are in substantial agreement with previous EXAFS analysis conducted on Ge-Se glasses^20^.

**Figure 4:**
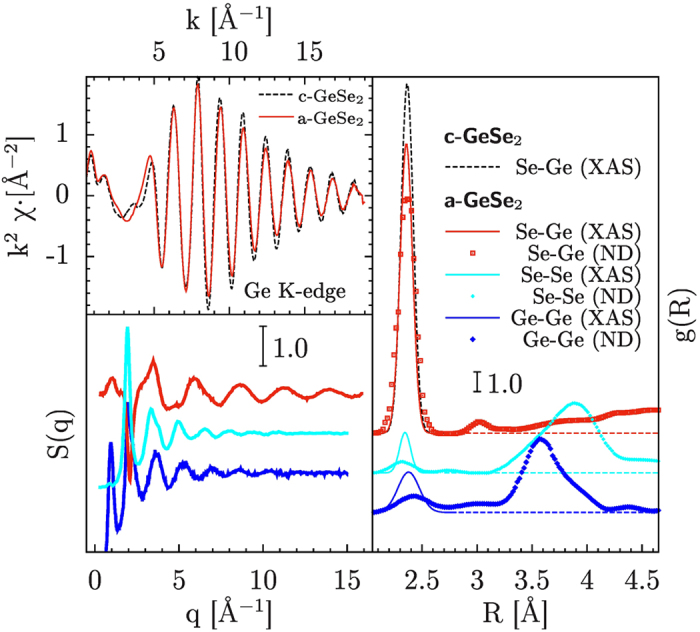
The XAS signals of c-GeSe_2_ (dots) and a-GeSe_2_ are compared in the upper-left panel, showing some differences (amplitude and near-edge) related to the different ordering of the two systems. The partial structure factors 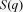 of a-GeSe_2_ measured by neutron diffraction^1^ are shown in the lower-left panel. The Se-Ge, Ge-Ge, and Se-Se partial distribution functions in a-GeSe_2_ as measured by XAS and neutron-diffraction (ND) are shown and compared with the Ge-Se distribution in crystalline GeSe_2_ (c-GeSe_2_) in the right hand-side panel.

A comparison with previous neutron-diffraction (ND) results^1^ on a-GeSe_2_ is reported in Fig. 4. In the right-hand side panel of Fig. 4 we report the partial pair distributions resulting by present XAS data-analysis, showing that present refinement is in substantial agreement with ND results. Due to the intrinsic short-range nature of the XAS probe, only first-neighbor 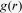 peaks (below 2.5 Å in Fig. 4) are accurately measured. In the present case, a direct proof of this statement can be obtained comparing the XAS signals of crystalline (dots) and amorphous GeSe_2_ as shown in the upper-left panel of Fig. 4. The Ge K-edge XAS signals differ slightly in amplitude due to a slight increase of structural disorder for first-neighbors in a-GeSe_2_, and some medium-range features can be seen in the c-GeSe_2_ spectra below 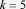 Å^−1^. On the other hand, the structure factor 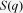 measured by ND (see lower-left panel in Fig. 4) is sensitive also to medium-range ordering in disordered systems. As discussed for example in Ref. 21, the strong photoelectron scattering amplitudes and short mean-free-path make XAS extremely sensitive to short-range ordering so that an improved structural refinement may be obtained combining ND and XAS data. Variations of the partial 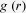
s below 2.5 Å have little effects in the 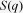 but strongly modify the XAS signals, so that the best-fit structural refinement mimics the ND results above 2.5 Å and the XAS ones below that distance. Present XAS structural refinement then confirms previous ND results about the presence of chemical disorder in GeSe_2_, indicating that the bond distributions of like-like and like-unlike first-neighbors are well-defined peaks associated with nearly-covalent bonding.

**Figure 5:**
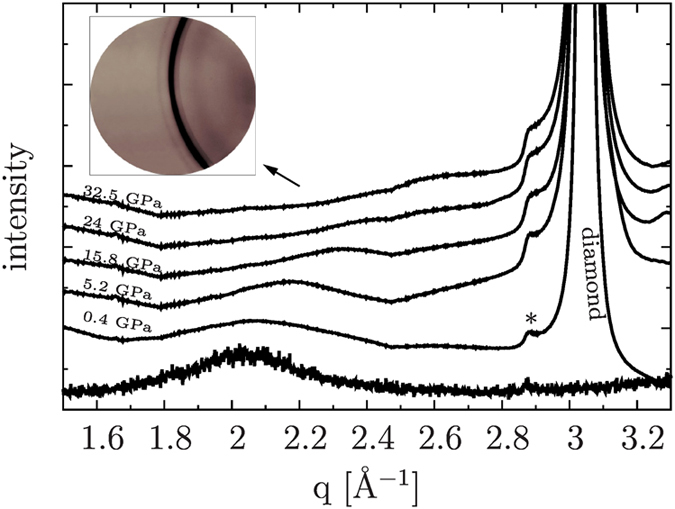
X-ray diffraction data for a-GeSe_2_ at ambient (bottom) and under high-pressure conditions (patterns are vertically shifted for clarity). In the inset we report a typical diffraction image, showing the intense ring from the nano-polycrystalline diamonds, a broad halo from the sample and two weak rings from the gasket (stainless steel), one of them being highlighted from the asterisk in the plot. The signal from the sample corresponds to the main peak of the structure factor, which is seen to shift to higher angles with the applied pressure. No sign of crystallization has been found up to 32.5 GPa.

As mentioned above, high-pressure Ge and Se K-edge XAS measurements up to about 30 GPa (see Fig. 1) were performed in dispersive mode. The amorphous character of the sample was checked simultaneously to the XAS measurements^13^ by means of the off-beam MAR detector. No sign of crystallization has been evidenced from the extracted diffraction patterns up to the maximum pressure of 32.5 GPa. A broad halo related to the GeSe_2_ sample is seen to shift to higher angles with the applied pressure, sign of a gradual compression of the interatomic distances, as shown in Fig. 5. The geometrical configuration of the experimental set-up for combined XAS and XRD did not allow to measure position and amplitude of the first sharp diffraction peak.

**Figure 6:**
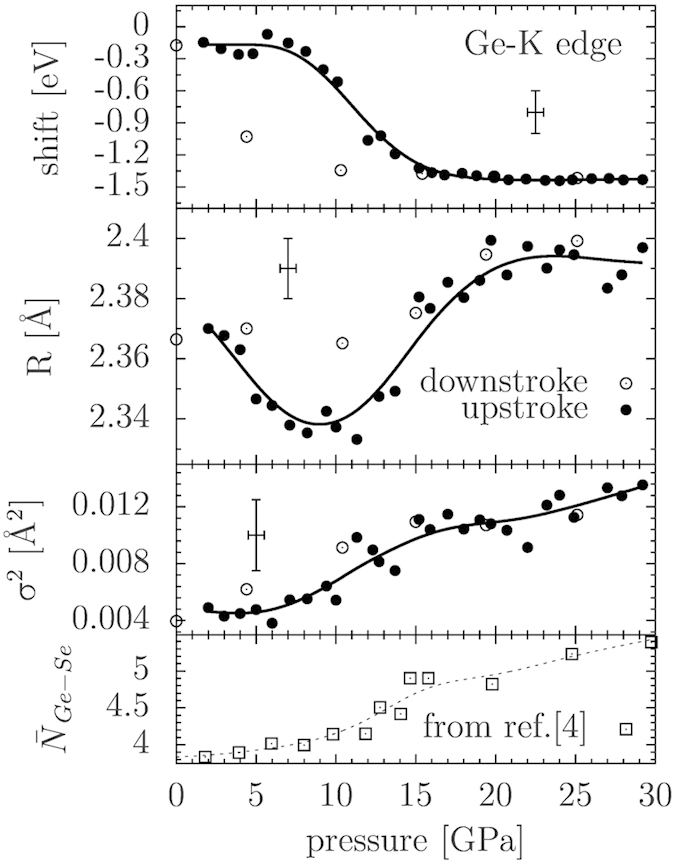
Trends of the Ge-K edge position, the first-shell Ge-Se distance and its mean square disorder as a function of pressure for a-GeSe_2_ from the double-edge refinements (see text). The average Ge-Se coordination number shown at the bottom of the figure was fixed for each pressure to the value reported by the molecular dynamics study performed in Ref. 4.

**Figure 7:**
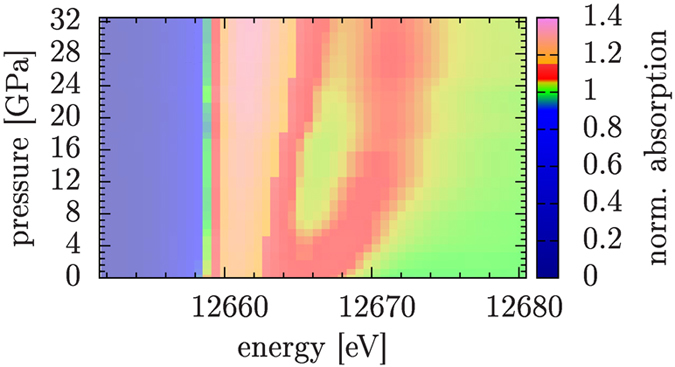
Variation of the Se K-edge XANES features as a function of pressure for a-GeSe_2_ (color scale reported in the right panel), associated with changes induced by compression in the local structure (see text).

The shape of the XANES (X-ray absorption near-edge structure) Ge K-edge spectra was observed to change with the applied pressure, showing a 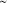
1.5 eV shift of the absorption edge to lower energies (see upper panel in Fig. 6), here interpreted as a sign of metallization. The shift of the edge is not observed at the Se K-edge, whose position is substantially not affected by pressure within the experimental uncertainty. In a disordered semiconductor like GeSe_2_ localization phenomena and different projected density of states are expected at Ge and Se sites, and those features can show different trends as a function of pressure, as discussed in Ref. 4 by electron structure calculations. Our results suggest that delocalized states near the Fermi energy start to be present at moderate pressure prevalently at the Ge sites. On the other hand, the Se K-edge XANES spectra show a clear trend as a function of pressure in the position of the first feature (shoulder) above the whiteline, as shown in Fig. 7. The Se K-edge XANES shoulder is seen to shift to higher energies of about 5 eV by increasing pressure up to 12 GPa. Such a modification is compatible with a compression of the Se-Se inter-layer distances (about 3.5 Å for the crystalline phase at ambient conditions) which can be estimated by means of the so-called Natoli’s rule^22^
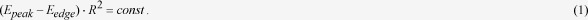
to be about 0.8 Å.

The changes in both Ge and Se K-edge XANES spectra can be assigned to a gradual charge transfer from intra-layer to inter-layer bonds. Such an interpretation is strengthened by the fact that at moderate pressures (below 10 GPa) we see a larger variation of the Se feature, which is more sensitive to the inter-molecular environment due to the position of the Se atoms at the border of the layers. This feature can be to some extent considered as a XAS analogous of the first sharp diffraction peak (FSDP), due to their similar behavior. In a previous work on GeS_2_^23^, the FSDP was seen to shift significantly from its initial position at an initial stage of compression, indicating a change in the intermediate range order. Such a phenomenon can be alternatively explained as the collapse of macroscopic voids naturally present in a disordered material. On the other hand, the principal peak was found to shift only at higher pressures, due to short-range changes. In the intermediate pressure range (10–15 GPa, see Fig. 6), the largest variation is seen in the Ge K-edge XANES, which is more directly connected to the intra-layer environment and therefore more sensitive to modifications occurring within the Ge-Se_4_ tetrahedra.

As a starting point to analyze the dispersive high-pressure EXAFS data, a double-edge analysis was performed on the same samples (crystalline and amorphous GeSe_2_) at ambient conditions with a traditional energy-scanning set-up, as mentioned above. Non-structural parameters such as the theoretical edge energy (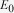) and the amplitude factor 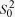 were kept fixed, minimizing the fluctuations of the structural parameters for increasing pressures. The same fitting strategy adopted for ambient pressure EXAFS data-analysis was applied for high-pressure structural refinement obtaining the trends shown in Fig. 6.

Due to the strong correlation among coordination numbers and disorder parameters, the structural refinement was performed assuming the known increasing trends of coordination numbers observed in diffraction experiments and obtained in molecular-dynamics (MD) simulations (see Ref. 4 and refs. therein). Furthermore, we assumed that no variation in chemical ordering was taking place upon increasing pressure. The short energy range available and the relatively high noise level is a well known drawback of the EXAFS spectra collected with the energy-dispersive technique, therefore quantitative information about coordination numbers and Ge-Ge and Se-Se bonding was not obtained in the present high-pressure work.

**Figure 8:**
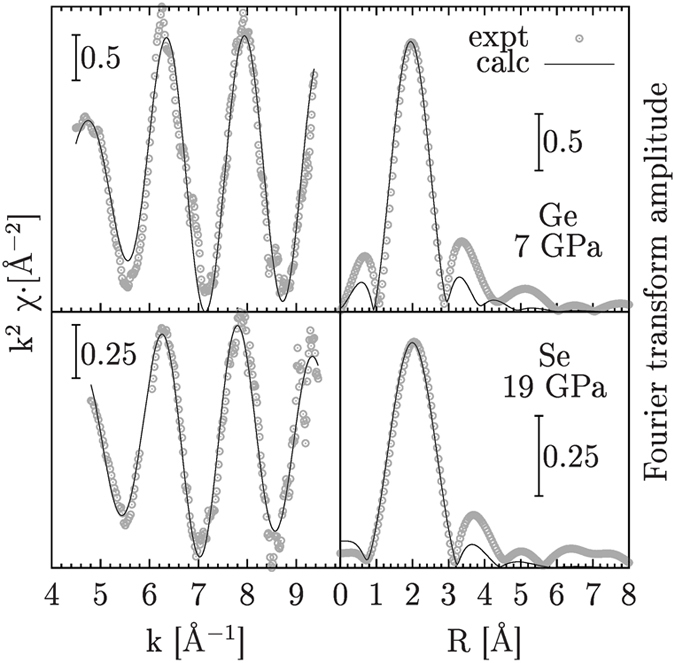
Left panels: Ge (upper inset) and Se (lower inset) K-edge XAS experimental (circles) and best-fit calculated structural signals of a-GeSe_2_ at high pressure (7 and 19 GPa respectively). Right-side panels: Fourier transform spectra of the XAS structural signals reported in the left-side panels. An excellent agreement is obtained modeling the first-neighbor Ge-Se distribution as reported in the text.

Several spectra were collected at each pressure point, by moving slightly the sample across the beam to average focusing imperfections, sample inhomogeneities and in order to increase the statistics. Given the optics of the experimental set-up for dispersive XAS, it was impossible to collect data simultaneously for the two edges: the data were therefore combined using spectra collected under similar thermodynamic conditions in separate experiments. For each pressure point the different spectra collected at the two edges were combined to give several sets of best-fit parameters and average values are presented in Fig. 6. Selected high pressure Ge and Se K-edge XAS spectra and their structural refinements are shown in Fig. 8. The good agreement between experimental and best-fit calculations observed in Fig. 8 demonstrates that reliable quantitative information about the first-neighbor distance distribution could be obtained under high pressure conditions.

As shown in Fig. 6, the first-neighbor Ge-Se average distance is first decreasing for increasing applied pressure up to about 10 GPa. The observed initial decrease in the first-shell distance is mainly associated with a compression of the less dense regions of the material, namely the inter-layer spacings. This is also connected to a gradual increase in the coordination number as predicted also by MD simulations^4^. The mean Ge-Se coordination was also previously estimated by XRD measurements to be 4.5 at 9.3 GPa^8^. In the present work, the Ge-Se average distance is found to increase above 10 GPa, at larger pressures than observed by XRD before^8^. A reasonable interpretation of this behavior is that the first densification stage exhausts the ‘free’ space available and brings the atoms close enough for the steric hindrance to play an important role. This in turn causes an increase in the first-shell average distances for pressures above 10 GPa in order to accommodate a further coordination increase. Molecular dynamics simulations for a-GeSe_2_ has shown the average germanium coordination to reach 5.5 at about 30 GPa^4^. The coordination increase is thus seen to take place at lower pressures than for GeS_2_^23^ (around 10 GPa instead of 15 GPa). The non-trivial indication is that the larger Se atoms can leave less space for the first compaction process, favoring the increase in mean first-shell distances and coordination number. Moreover, the material is found to be always amorphous with a disordered first-neighbor distribution, during the entire pressurization process, as shown by the 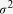 bond variance reported in Fig. 6 and by our XRD results. The variance increases as expected when further atoms has to be accommodated in the first-neighbor shell, breaking the more ordered and symmetric fourfold coordination (Ge sites). In particular, we interpreted this increase as a spatially inhomogeneous conversion to higher coordination environments, which therefore average out to dampen the EXAFS signal. The fact that the bond variance (mean square relative displacement) is found to be so high even at the larger pressures attained is seen as an evidence that the conversion to the octahedral configuration is not yet complete. This result is in agreement with previous MD simulations^4^, predicting a full conversion to octahedral coordination only at pressures around 50 GPa. Generally speaking, the compaction mechanism seems to be analogous to what happens in similar systems such as GeO_2_ and GeS_2_^23^,^24^. Finally, our results concerning the Ge-Se average distance under pressure seem to be in agreement with a recent neutron diffraction study performed on glassy GeSe_2_^25^ up to 16 GPa.

As a concluding observation, a hysteretic behavior is observed for both the first-neighbor average distance and the Ge K-edge energy in Fig. 6, again in analogy with similar tetrahedral-like systems. The local structure tends to retain the main features of the high-pressure regime, therefore the upstroke path is not followed exactly along the downstroke. However, no evidence of permanent densification is identified, and the initial values of edge energy and first-neighbor parameters are recovered within the experimental uncertainty.

## Discussion

We performed an accurate two-edges XAS study of a-GeSe_2_ under ambient and high-pressure conditions. Multiple-edge XAS data-analysis of low-noise data of c-GeSe_2_ and a-GeSe_2_ at ambient conditions allowed us to refine the short-range structure of a-GeSe_2_, reconstructing the first-neighbor distribution function that was found to be in substantial agreement with previous neutron diffraction measurements. Presence of chemical disorder was taken into account and the inclusion of about ~20% wrong bonds was found to be statistically significant in order to improve the quality of the data reduction.

The *in situ* high pressure study was performed combining dispersive XAS and XRD measurements up to 32.5 GPa. The sample is found to remain amorphous up to the highest pressures attained, and a reversible red-shift of the Ge K-edge energy, of about 1.5 eV, is observed above 10 GPa up to about 15 GPa. This shift is interpreted as the signature of a metallization process associated with the appearance of a delocalized electron states prevalently around the Ge sites. The XAS structural refinement allowed us distinguish two compression stages: i) a first one involving a decrease of the first-neighbor distances up to about 10 GPa, in the same pressure region of the breakdown of the intermediate-range order previously observed by XRD^8^; ii) the second one characterized by the increase of the first-shell average distance and bond fluctuations, and of the coordination number. This second stage is related to a reversible non-isostructural transition involving a gradual conversion from tetra- to octa-hedral geometry which is not found to be fully completed at pressures up to 30 GPa.

Some questions are still left open by the present study. For example, future efforts may be devoted in searching a pressure limit for the conversion to a full octahedral symmetry, or reaching a possible crystallization onset. Another interesting question concerns changes in chemical disorder upon pressurization, that are likely to be present in such systems but can not be put to a test in view of the limited energy extension of the dispersive XAS data. In this regard, shedding light on chemical disordering under pressure could be possible by high-quality double-edge XAS data refinement using traditional scanning-energy micro-focused XAS on DACs.

## Methods

The amorphous sample used for the high pressure measurements has been purchased from Sigma-Aldrich (CAS number 12065-11-1, 99.999% purity). The crystalline counterpart have been synthesized by annealing for 12 h at 600 °C the same sample previously sealed under vacuum into appropriately designed quartz vessels. Both crystal and amorphous phase have been checked by XRD.

High-quality Ge and Se K-edge XAS spectra of crystalline (c-GeSe_2_) and amorphous (a-GeSe_2_) germanium diselenide were measured under ordinary pressure conditions at the XAFS beamline at ELETTRA (Trieste, Italy) synchrotron radiation facility^11^. Finely ground powder was compressed into standard pellets using cellulose as inert matrix into appropriate ratio to ensure good absorption contrast for the different edges.

Energy dispersive XAS data at high pressure were acquired at the ODE beamline (SOLEIL synchrotron, Saclay, France, see Ref. 12) at both Ge and Se edges in two separate runs. The diamond anvil cell (DAC) used was equipped with nano-polycrystalline diamonds^14^ (NPD) and silicone oil was used as pressure transmitting medium, with a ruby chip as pressure marker. The energy was calibrated by comparing the spectra from two reference foils to traditional energy scanning data and by fitting with a cubic function. The focal spot was about 50 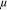
m full-width half-maximum, whit a gasket hole 150 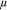
m wide. Simultaneous angle-dispersive XRD data were collected by means of a MAR detector put slightly off the beam axis, calibrated in turn by fitting the diffraction peaks from a reference LaB_6_ sample. The energy (12626.5 eV) was selected by closing the exit slits of the polychromator to be just below the selenium absorption edge, in order reduce the scattering background^13^.

The quality of the collected spectra is good as shown in Fig. 1. The smoothness of the data is mainly due to the use of NPD diamonds, which allows for an easier alignment process not requiring DAC rotation/tilting to remove the vexing Bragg reflections from diamonds normally present in this energy range^15^. Nevertheless, some weak although sharp features can be anyway seen in the absorption background at the Se K-edge, which may be related to an imperfect iso-orientation of the nanocrystallites in the diamonds but which are more probably connected to an imperfect glitch compensation due to the optics.

## Additional Information

**How to cite this article**: Properzi, L. *et al.* Short-range order of compressed amorphous GeSe_2_. *Sci. Rep.* **5**, 10188; doi: 10.1038/srep10188 (2015).
